# Phylogeography and Sex-Biased Dispersal across Riverine Manatee Populations (*Trichechus inunguis* and *Trichechus manatus*) in South America

**DOI:** 10.1371/journal.pone.0052468

**Published:** 2012-12-20

**Authors:** Paula Satizábal, Antonio A. Mignucci-Giannoni, Sebastián Duchêne, Dalila Caicedo-Herrera, Carlos M. Perea-Sicchar, Carmen R. García-Dávila, Fernando Trujillo, Susana J. Caballero

**Affiliations:** 1 Laboratorio de Ecología Molecular de Vertebrados Acuáticos-LEMVA, Departamento de Ciencias Biológicas, Universidad de los Andes, Bogotá, Colombia; 2 Puerto Rico Manatee Conservation Center, Inter American University of Puerto Rico, Bayamón, Puerto Rico; 3 School of Biological Sciences, The University of Sydney, Sydney, Australia; 4 Fundación Omacha, Bogotá, Cundinamarca, Colombia; 5 Dirección Regional de la Producción de Loreto, Iquitos, Perú; 6 LBGM, Instituto de Investigaciones de la Amazonía Peruana, Iquitos, Perú; Ecole Normale Supérieure de Lyon, France

## Abstract

Phylogeographic patterns and sex-biased dispersal were studied in riverine populations of West Indian (*Trichechus manatus)* and Amazonian manatees (*T. inunguis*) in South America, using 410bp *D-loop* (Control Region, Mitochondrial DNA) sequences and 15 nuclear microsatellite loci. This multi-locus approach was key to disentangle complex patterns of gene flow among populations. *D-loop* analyses revealed population structuring among all Colombian rivers for *T. manatus,* while microsatellite data suggested no structure. Two main populations of *T. inunguis* separating the Colombian and Peruvian Amazon were supported by analysis of the *D-loop* and microsatellite data. Overall, we provide molecular evidence for differences in dispersal patterns between sexes, demonstrating male-biased gene flow dispersal in riverine manatees. These results are in contrast with previously reported levels of population structure shown by microsatellite data in marine manatee populations, revealing low habitat restrictions to gene flow in riverine habitats, and more significant dispersal limitations for males in marine environments.

## Introduction

Differences in dispersal behavior between males and females can have a profound effect on population dynamics. Male-biased dispersal (MBD) has often been described in mammals [1], [2], [3], and has been proposed for manatees, where females are philopatric and males migrate, implying gene flow among populations [4], [5]. Male driven gene flow could be a strategy against male competition for females, inbreeding and resource competition [6].

Studies that include telemetry and field observations on West Indian (*Trichechus manatus*) and Amazonian (*T*. *inunguis*) manatees have revealed seasonal and water level dependent migration [5], [7], [8], [9], [10]. These movements seem to be driven by changes in water temperature, food availability and space [5], [8]. However, there is evidence for site fidelity in individuals, females showing smaller home ranges than males [5]. Reproductive migrations have also been reported for manatee promiscuous mating system, where adult males move long distances escorting estrous females, forming temporary mating herds [4], [11], [12].

Population genetics is a useful tool for understanding male and female population dynamics of West Indian and Amazonian manatees. However, low sampling due to cryptic behavior has limited the definition of manatee population structure. Using multiple unlinked loci for populations with smaller sampling sizes improves accuracy in population genetics estimates [13], [14], [15], and provides a solution to study population dynamics in manatee populations. Primers for amplification of highly polymorphic bi-parentally inherited nuclear microsatellite loci have been developed for the Florida manatee [16], [17], [18], and effectively used to study manatee population structure in Florida, Puerto Rico, Belize and Mexico [19], [20], [21].

Previous studies using maternally inherited *D-loop* (Control Region, mitochondrial DNA) indicated high levels of structuring between *T. manatus* populations, showing three main haplotype clusters [22], [23], and one panmictic population for *T. inunguis* in Brazil, with high genetic variability and possible demographic expansion [23], [24]. Microsatellite data supported population division between Florida and Puerto Rican populations [21], the wetlands systems of the Gulf of Mexico and the Caribbean coast of Mexico [20], as well as between Belize City Cayes and the Southern Lagoon system in Belize [19].

In this study we tested the MBD hypothesis for riverine West Indian manatees in Colombia, where populations are distributed mainly in rivers and swamps [25], and Amazonian manatees in Colombia and Peru, by comparing levels of population structuring between *mtDNA D-loop*, and 15 microsatellite loci. These results have implications in assessing population migratory limits, which could help to define effective conservation units and enhance global and local management action plans to effectively protect manatee populations of both species.

## Materials and Methods

### Ethics Statement (Animal Research)

The sample collection methodology for this project was approved by Universidad de los Andes, Faculty of Sciences Ethics Comitee and was done following the Caribbean Stranding Network protocols, avoiding animal suffering.

Samples were collected as part of Colombia’s West Indian and Amazonian manatees National Management Plan. They were provided by Omacha Foundation (Colombian non-governmental organization), and were collected by employees from Colombian Regional Environmental Authorities (Corporaciones Autónomas regionales- CARs) during manatee rehabilitation and releasing programs over the past 20 years. Peruvian samples were collected by the PRODUCE - DIREPRO (regional authority) and were analyzed at the Peruvian Amazon Research Institute (IIAP) in Iquitos. Regional Environmental Authorities in Colombia and Perú do not require to obtain collection permits for particular projects (Ministry of Environment: Decreto 309 de 2000, Artículo 2, Párrafo 1 (Legislation attached)), as these institutions belong to the National Environmental Systems of the Ministry of Environment (Sistema Nacional Ambiental in Colombia).

Fecal samples were collected over the water column from wild individuals. Bone samples were obtained from manatee carcasses illegally killed by local fisherman, and seized by regional authorities. Blood and tissue samples were collected from live rescued individuals; blood was obtained from the anterior pectoral flipper, using a 1.5-inch needle, while tissue was collected from the tail fin using approved protocols.

### Sample Collection and DNA Extraction

Skin, blood and fecal samples were collected from wild and captive manatees. Bone, skin, and muscle samples were collected from carcasses. Skin, muscle and feces were preserved in 70% ethanol, and blood samples in EDTA lysis buffer [10 mM NaCl, 100 mM EDTA, 100 mM Tris (pH 8), and 1% (w/v) SDS]. A total of 97 samples were collected from 39 for *T. inunguis* and 58 for *T. manatus*.

**Figure 1:**
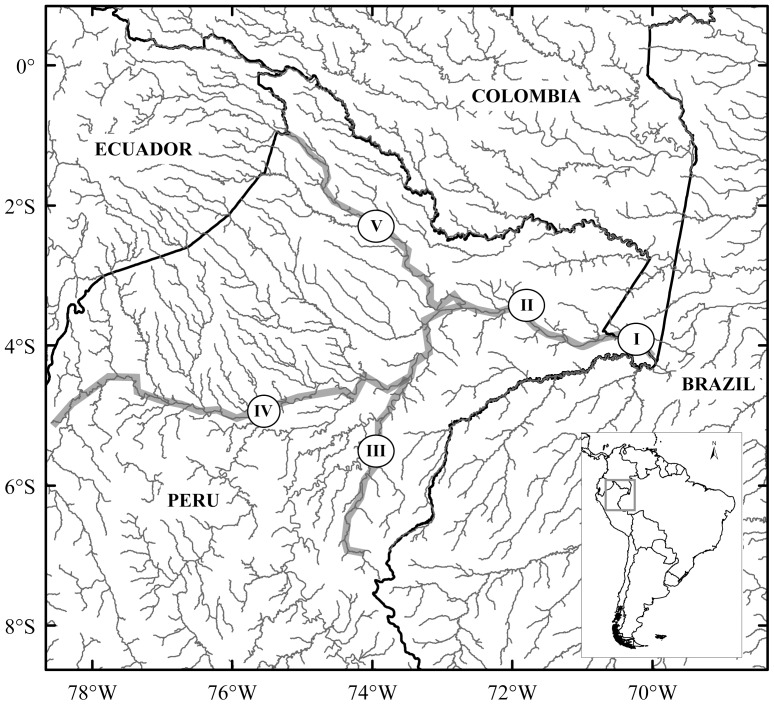
Map of five *T. inunguis* sampled rivers. (I) Colombian Amazon, (II) Peruvian Amazon, (III) Ucayali, (IV) Marañón, and (V) Napo.

**Figure 2:**
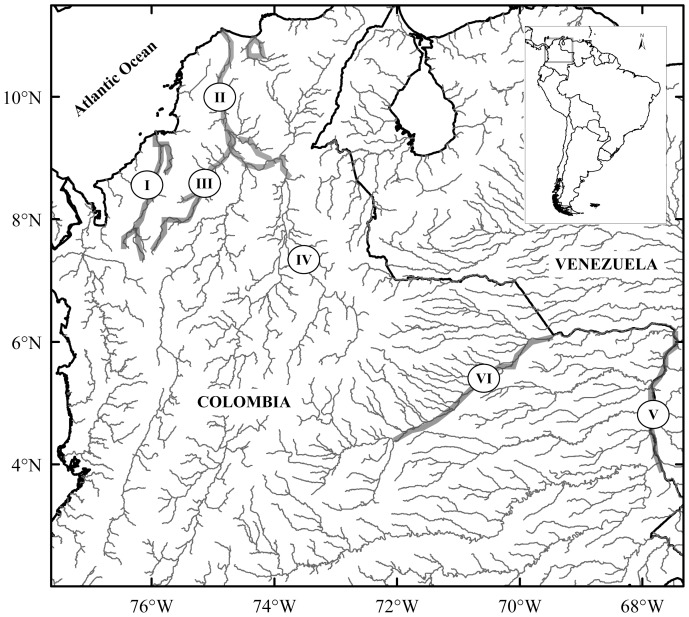
Map of six *T. manatus* sampled rivers. (I) Sinú, (II) Northern Magdalena, (III) San Jorge, (IV) Magdalena’s Ciénaga de Paredes, (V) Meta, and (VI) Orinoco.

Amazonian manatees were sampled from four Amazon tributaries, and five specific locations: (I) Colombian Amazon River (*n* = 6), (II) Peruvian Amazon River (*n* = 7), (III) Ucayali River (*n* = 13), (IV) Marañón River (*n* = 9), and (V) Napo River (*n* = 5) (Figure 1). West Indian manatees were sampled from six Colombian distinct locations: (I) Sinú River (*n* = 17), (II) Northern Magdalena River (*n* = 19), (III) San Jorge River (*n* = 10), (IV) Magdalena River’s Ciénaga de Paredes (*n* = 7), (V) Meta River (*n* = 2), and (VI) Orinoco River (*n* = 3) (Figure 2). Northern Magdalena River was considered a different sampling location from Magdalena River’s Ciénaga de Paredes (marsh), as it is located in the middle Magdalena basin, and is not directly connected to the Magdalena River. DNA extractions were performed using a phenol-chloroform protocol [26].

### PCR Analysis and Sequencing

A 410bp *D-loop* hypervariable portion of the *mtDNA CR* was amplified by the polymerase chain reaction (PCR), using two pairs of primers, depending on DNA quality. Good quality samples were amplified with CR4 and CR5 primer pair [22], each PCR mix of 30 µl reaction contained 1X reaction buffer (10 mM Tris–HCl, 50 mM KCl, pH 8.3), 1.5 mM MgCl_2_, 200 µM dNTP, 2 units BSA, 0.5 µM of each primer, and 1 U Biolase DNA polymerase (Bioline USA). PCR amplification conditions were: 94°C for 2 min, 34 cycles of 94°C for 30 s, 55°C for 45 s and 72°C for 40 s, with a final extension period of 72°C for 10 min. Low quality samples (faeces and bones) were amplified using LTMCR01 and HDDCR01 primer pair and previously published PCR conditions [27]. Volume reaction mixture contained 10µl Maxima® HotStart PCR Master Mix (Fermentas), and 0.5 µM of each primer. All sample purification and DNA sequencing was completed by Macrogen Inc. (Seoul, South Korea) on a 3730xl DNA Analyzer (Applied Biosystems).

Fragment analysis of 15 polymorphic microsatellite loci (*TmaKB60, TmaA02, TmaE01, TmaE02, TmaE07, TmaE08, TmaE11, TmaE26, TmaH13, TmaJ02, TmaK01, TmaF14, TmaM79, TmaSC5, TmaSC13*) [16], [17], was achieved following Kellogg [28] PCR conditions, and visualized on an Applied Biosystems ABI 3100 Genetic Analyzer (Universidad de los Andes). All individuals were genotyped for at least 12 loci. Allelic dropout was tested by randomly repeating fragment analysis for 10 homozygote samples per locus.

### Sequences Statement

New haplotypes reported in this manuscript have been deposited in GenBank under the accession numbers: [JX982639: JX982651].

### Data Analysis

410bp *D-loop* sequences and previously reported haplotypes available in Genbank (Accession numbers [AY963840: AY963893], [AY738549: AY738579]) [22], [23], [24], were aligned manually using MacClade version 4.08 [29]. *T. inunguis* sequences were analyzed using two approaches: the first included 410bp sequences from Colombian and Peruvian rivers, and the second reduced the length of sequences to 361bp, to include sequences and geographic information of Brazilian rivers (Japurá, Tefé, Negro, Solimões, Amazonas, Pará) obtained from Cantanhede *et al*. 2005. The number of haplotypes (*h*), nucleotide (*π*), and haplotype (*Hd*) diversity indices for each population [30], were estimated in ARLEQUIN version 3.5 [31], and DNASP version 5.10.01 [32]. Haplotype networks were constructed using the Median Joining network (MJN) algorithm in NETWORK version 4.6.1.0 [33].

ARLEQUIN was used to estimate the number of alleles per locus (*N_A_*), levels of polymorphism shown by microsatellite loci, departures from Hardy-Weinberg equilibrium (HWE) (Markov chain 100000, dememorization steps 100), and linkage disequilibrium (LD) (permutations 10000). *P* values were adjusted using Bonferroni correction for multiple comparisons.

Presence of null alleles was evaluated by comparing expected and observed heterozygosities with MICRO-CHECKER version 2.2.3 with Bonferroni correction [34], followed by allele randomization (10000 repetitions) within each population and locus using custom R code version 2.14.2 [35] (R Script S1: available as supporting information). In order to test the effect of including possible loci with null alleles a locus bootstrapping was performed using custom code in R (R Script S2: available as supporting information). This method is based on randomly sampling loci with replacement, and rerunning analysis of population structure. Specifically, null alleles cause lower heterozygosity than expected under random mating, therefore, artificially high population differentiation. If our estimates of population structure are driven by null alleles at any single locus then its exclusion from the dataset in bootstrap replicates will reveal lower population structure than that of the original dataset.

Independent genealogies were obtained in a Bayesian framework for microsatellite loci per species, assuming a birth death speciation process [36], as implemented in BEAST version 1.7.1 [37]. The settings were 100 million MCMC chain lengths, with a sampling frequency of 1000. MCMC convergence was diagnosed using TRACER version 1.5 [38], by verifying that ESS (estimated sample size) for all parameters were at least 200. Sampled trees were then summarized into a highest clade credibility tree, discarding 10 million steps as burn-in with TREEANNOTTATOR version 1.7.1 (Drummond & Rambaut 2007), and drawn in FIGTREE version 1.3.1 [39]. Populations monophyly on each genealogy was assessed using custom R code (R Script S3: available as supporting information) and the package APE version 3.0–2 [40].

STRUCTURE version 2.3.3 [41], was used to identify genetic subdivision. Ten independent simulations per population cluster values (*K*) from 1 to 10, were run without an *a priori* population assignment, using the LOCPRIOR prior to improve the detection of weak population structure [42]. Simulations were performed under an admixture model with 1million repetitions of Monte Carlo Markov Chain (MCMC) and a burn-in of 100000 steps. The number of clusters was estimated and summarized using ad hoc statistic Delta*K* [43] implemented in STRUCTURE HARVESTER web version 0.6.92 [44], and variance among runs was estimated using CLUMPP version 1.1.2 [45].

Among and within population subdivision for *D-loop* and microsatellite loci were estimated using Wright’s fixation index *F_ST_,* inbreeding coefficient *F_IS_*, and molecular variance (AMOVA) [31], [46] in ARLEQUIN. A correlation significance test between *F_ST_* pairwise distance matrix for mtDNA and microsatellite loci was performed using a Mantel Test under Spearman (non-parametric) method, with 100000 permutations using the VEGAN package in R [47].

Migration rates among populations were estimated using a Bayesian inference coalescent approach in MIGRATE-N version 3.2.17 [48]. Four migration models were tested for *T. inunguis*, and five for *T. manatus* according to possible geographic barriers:

- *T. inunguis*, M_0_: No migration among populations, M_1_: One single panmictic population, M_2_: Full symmetric migration among all populations, M_3_: [Colombian Amazon – Peruvian Amazon], [Peruvian Amazon – Ucayali], [Ucayali – Marañón], [Peruvian Amazon – Napo], [Peruvian Amazon – Marañón].
- *T. manatus*, M_0_: No migration among populations, M_1_: One single panmictic population, M_2_: Full symmetric migration among all populations, M_3_: [Sinú – Northern Magdalena – San Jorge – Magdalena’s Ciénaga de Parades], [Meta – Orinoco], M_4_: [Sinú – San Jorge], [Sinú – Northern Magdalena], [Northern Magdalena – San Jorge], [Northern Magdalena – Magdalena’s Ciénaga de Parades], [Meta – Orinoco].

A total of three independent analyses with chain length of 100000000 were performed for each migration model to ensure sampling from the stationary distribution. Convergence of the MCMC was assessed through visual inspection of the all parameter traces. In all cases, every parameter had an effective sample size of at least 300. The best-fitting migration model for each species was chosen based on the Bayes factors of the harmonic mean of the likelihood (for an example of this method see Beerli & Palczewski, 2010 [49]), used as an approximation of the marginal likelihood of the model [50]. Models were first ranked according to their likelihood harmonic means, and then the Bayes factors for every model vs. that with the highest likelihood were calculated.

## Results

### Mitochondrial Genetic Diversity

**Figure 3:**
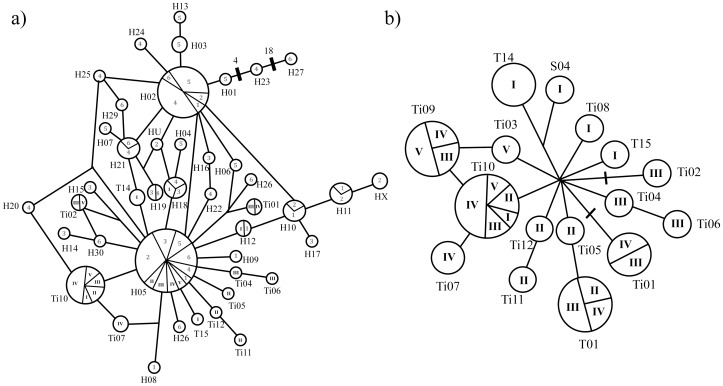
*T. inunguis* Median Joining network (MJN). a) 361bp MJN of Colombian, Peruvian populations, and previously published Brazilian populations [24]. b) 410bp MJN of Colombian and Peruvian manatees. Both MJN revealed one *D-loop* haplotypes cluster for Amazonian manatees (see **Figure 1** for Colombian and Peruvian population name abbreviations; Brazilian populations are abbreviated following Cantanhede *et al.* 2005∶1-Japurá, 2-Tefé, 3-Negro, 4-Solimões, 5-Amazonas, 6-Pará)). Haplotypes names are written outside of haplotypes pie charts, while population names are written inside.

**Figure 4:**
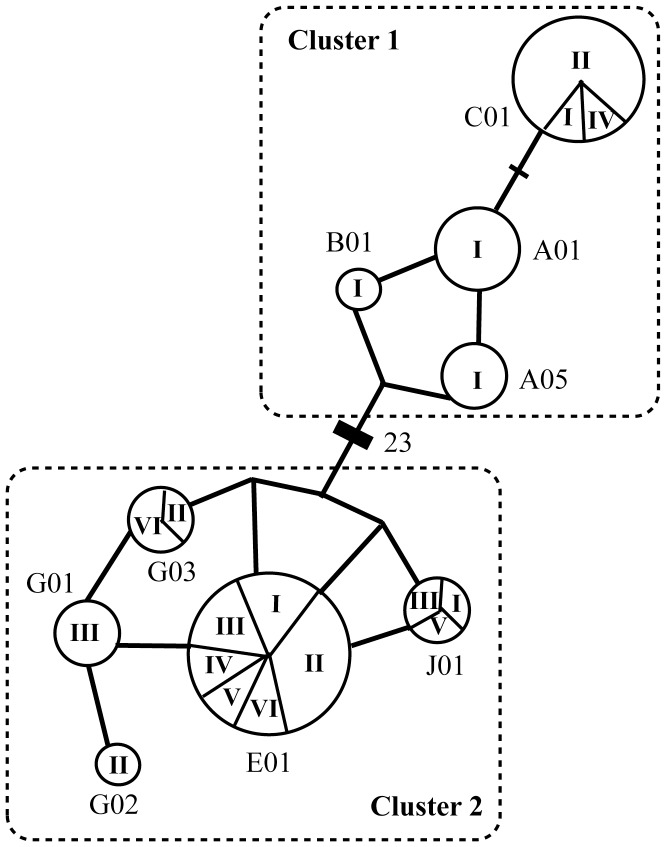
*T. manatus* Median Joining network (MJN). The MJN reveals two *D-loop* haplotypes clusters of Colombian manatees (see **Figure 2** for population name abbreviations). Haplotypes names are written outside of haplotypes pie charts, while population names are written.

Sixteen 410bp *D-loop* haplotypes were found for Colombian and Peruvian Amazonian manatees, including 12 new haplotypes (*Ti01-Ti12*) (Figure 3), and nine haplotypes for Colombian West Indian manatees, including one new haplotype (*G03*) (Figure 4). New haplotype sequences were submitted to Genbank under accession numbers: [JX982639: JX982651]. When assigning haplotype redundancy using 361bp sequences for *T. inunguis,* haplotypes *Ti02* and *Ti03* were reduced to one haplotype (*Ti02*), haplotype *Ti08* was redundant to haplotype *H18*, haplotypes *Ti09* and *T01* were redundant to haplotype *H05*, and haplotype *S04* was redundant to haplotype *H12*. Average *π* among *T. inunguis* individuals when using 410bp sequences was 0.472% (standard deviation (SD) 0.055%), and overall *Hd* was 0.885 (SD 0.035), showing the highest diversity in the Colombian Amazon population (Table 1). When using 361bp sequences, the average *π* was 0.718% (SD 0.113%), the overall *Hd* was 0.903 (SD 0.019), and the highest diversity was found in the Pará River. *T. manatus* sequences were more diverse than those of *T. inunguis* when using 410bp sequences, with an average *π* of 3.018% (SD 0.315%), and overall *Hd* of 0.76 (SD 0.052), and less diverse when compared with 361bp sequences that included Brazilian Rivers. The Sinú River’s West Indian manatees had the highest mitochondrial diversity (Table 1).

**Table 1:** *D-loop* number of haplotypes (*h*), nucleotide (*π*), and haplotype (*Hd*) diversity indices with 95% confidence intervals, and 15 microsatellite loci estimates of number of alleles per locus (*N_A_*), mean observed heterozygosity (*H_O_*), and mean expected heterozygosity (*H_E_*), for *T. inunguis*, and *T. manatus* populations.

	*T. inunguis*					*T. manatus*					
	Colombian Amazon	Peruvian Amazon	Ucayali	Marañón	Napo	Sinú	San Jorge	Northern Magdalena	Magdalena’s Ciénaga de Paredes	Meta	Orinoco
Samples *D-loop*	6	6	12	9	5	13	8	18	3	2	3
*h*	6	3	10	3	4	6	3	4	2	2	2
π (%)	0.602±0.435	0.293±0.249	0.536±0.360	0.244±0.205	0.489±0.383	3.455±1.860	0.192±0.176	3.397±1.789	4.390±3.380	0.243±0.345	0.325±0.334
*Hd*	0.933±0.122	0.800±0.172	0.894±0.078	0.750±0.112	0.900±0.161	0.833±0.071	0.679±0.122	0.628±0.073	0.667±0.314	1.000±0.500	0.667±0.314
Samples microsatellite loci	5	6	13	9	5	13	8	18	7	2	3
*Mean N_A_*	4.333±1.718	3.667±1.345	5.733±1.751	4.533±1.685	4.067±1.387	7.067±2.434	5.467±2.066	7.533±2.232	4.600±1.404	2.500±0.650	3.286±1.139
*Mean H_O_*	0.420±0.367	0.574±0.264	0.450±0.284	0.496±0.297	0.543±0.253	0.443±0.353	0.373±0.270	0.471±0.268	0.338±0.269	0.500±0.393	0.429±0.304
*Mean H_E_*	0.731±0.176	0.696±0.168	0.647±0.236	0.609±0.208	0.749±0.110	0.782±0.131	0.748±0.193	0.778±0.098	0.761±0.126	0.714±0.152	0.724±0.181

As previously shown by Cantanhede *et al.* 2005, and Vianna *et al.* 2006, one diverse Median Joining Network (MJN) cluster was obtained for *T. inunguis D-loop* haplotypes using both 361bp and 410bp approaches, with all haplotypes being closely related among them (Figure 3). In contrast to *T. inunguis, T. manatus* populations contained haplotypes from two MJN clusters, which differ by at least 23 mutations, and corresponded to previously reported clusters obtained by García-Rodríguez *et al.* 1998 and Vianna *et al.* 2006. San Jorge, Meta and Orinoco rivers *T. manatus* haplotypes were only present in cluster 2 (Figure 4).

### Microsatellite Genetic Variation

All 15 microsatellite loci had between 5 and 12 alleles per locus for *T. inunguis,* and between 4 and 13 for *T. manatus*. Both species showed high allelic diversity and low levels of heterozygosity, with observed heterozygosity (*H_O_*) lower than expected heterozygosity (*H_E_*) per locus per population (Table 1). Allelic dropout was dismissed since no significant differences among the genotypes observed when randomly repeating homozygotes individuals were detected. No linkage disequilibrium was observed, and the majority of loci per population presented deviations from HWE at least for one population (except *T. inunguis*: *TmaKB60, TmaE02, TmaSC13, TmaSC5*; and *T. manatus: TmaSC05*).

We found evidence for presence of null alleles in all loci in *T. manatus,* and 8 loci in *T. inunguis* (*TmaA02, TmaE01, TmaE07, TmaE08, TmaE26, TmaH13, TmaJ02, TmaM79*) in at least one population. Differences in levels of population structure were found for each allele bootstrap replicate. Moreover allele randomization revealed higher heterozygote frequencies in comparison with observed values on every locus per population, due to homozygote excess.

### Population Structure

Populations were not monophyletic in any of the microsatellite loci genealogies trees in either species. The amount of population clusters according to STRUCTURE after using ad hoc statistic Delta*K*, and variance among runs, was *K* = 2 in both species. Neither of the clusters obtained were consistent with sampled populations. The two clusters shown for *T. manatus* were not correlated with the two *D-loop* clusters, showing not only differences in levels of structure among microsatellites loci and mitochondrial *D-loop*, but also in their evolutionary trajectory.

Estimates of *F_IS_* for *T. manatus* and *T. inunguis* were significant (*P*<0.05), indicating high levels of inbreeding within populations at 0.456 and 0.282, respectively. Differences among populations using 410bp *D-loop* sequences demonstrated structure between Peruvian and Colombian *T. inunguis* manatees (*φ_ST_* = 0.151, *P* = 0.039), being lower but significant with microsatellite data (*φ_ST_* = 0.080, *P*<0.001) (Table 2). The structure among these groups is not strongly evident in the network. However, it is possible to identify that Colombian Amazon only shares one haplotype (*Ti10*) with Perú, while the rest of its haplotypes are unique for this population, contrasting with Peruvian populations which share more than one haplotype among them (Figure 3b).

**Table 2:** Pairwise *F_ST_* and *P* values estimates for five *T. inunguis* populations.

	Colombian Amazon*	Peruvian Amazon*	Ucayali	Marañón	Napo*
Colombian Amazon*		0.108	0.12	0.11	0.226
		*P* = 0.225±0.004	*P* = 0.021±0.002	*P* = 0.024±0.002	*P* = 0.027±0.002
Peruvian Amazon*	0.129		−0.031	−0.031	0.009
	*P* = 0.005±0.001		*P* = 0.668±0.004	*P* = 0.473±0.005	*P* = 0.391±0.005
Ucayali	0.113	0		0.064	0.139
	*P* = 0.002	*P* = 0.821±0.004		*P* = 0.090±0.003	*P* = 0.037±0.002
Marañón	0.127	0.01925	0.022		0.163
	*P*<0.001	*P* = 0.302±0.005	*P* = 0.160±0.004		*P* = 0.082±0.002
Napo*	0.074	0.046	0.021	0.016	
	*P* = 0.043±0.002	*P* = 0.176	*P* = 0.406±0.005	*P* = 0.469±0.005	

Above diagonal for *D-loop*, and bellow diagonal for microsatellite data.

* Comparisons between these populations were obtained with few samples.

+ Few samples for *D-loop* data.

When including 361bp *D-loop* sequences, population structure was found displaying two main groupings, a Peruvian population including all Peruvian tributaries to the Amazon River and a Colombian and Brazilian population that included the Colombian portion of the Amazon River and all other rivers and tributaries of Brazil (*φ_ST_* = 0.188, *P*<0.001).

In Colombian *T. manatus* structure levels were even higher, revealing five different populations: Sinú River, Northern Magdalena River, San Jorge River, Magdalena River’s Ciénaga de Paredes, and Meta and Orinoco rivers (*φ_ST_* = 0.349, *P*<0.001), or four groups if excluding Meta and Orinoco due to low sampling sizes (Meta: *n* = 2, Orinoco: *n* = 3) (Table 3). However, no significant differences were found between locations for microsatellite data.

**Table 3:** Pairwise *F_ST_* and *P* values estimates for six *T. manatus* populations.

	Sinú	San Jorge	Northern Magdalena	Magdalena’s Ciénaga de Paredes+	Meta*	Orinoco*
Sinú		0.496	0.050	−0.047	0.315	0.378
		*P = *0.002±0.001	*P = *0.155±0.004	*P = *0.306±0.004	*P = *0.193±0.004	*P = *0.074±0.002
San Jorge	0.043		0.244	0.309	0.033	0.271
	*P*<0.001		*P = *0.026±0.002	*P = *0.157±0.004	*P = *0.302±0.004	*P = *0.155±0.004
Northern Magdalena	0.021	0.022		−0.230	0.039	0.128
	*P = *0.010	*P = *0.031±0.002		*P = *0.999	*P = *0.234±0.003	*P = *0.240±0.004
Magdalena’s Ciénaga de Paredes+	0.073	0.054	0.057		−0.200	0.022
	*P*<0.001	*P = *0.003±0.001	*P*<0.001		*P = *0.999	*P = *0.401±0.004
Meta*	0.092	0.129	0.099	0.173		0.340
	*P = *0.008±0.001	*P = *0.009±0.001	*P = *0.004±0.001	*P*<0.001		*P = *0.290±0.005
Orinoco*	0.078	0.034	0.056	0.033	0.140	
	*P = *0.003±0.001	*P = *0.122±0.003	*P = *0.014±0.001	*P = *0.134±0.003	*P = *0.009±0.001	

Above diagonal for *D-loop*, and bellow diagonal for microsatellite data.

* Comparisons between these populations were obtained with few samples.

+ Few samples for *D-loop* data.

The Mantel test presented no correlation between pairwise *F_ST_* distance matrixes for either species (*T. inunguis*: r = 0.268, *P* = 0.383, and *T. manatus*: r = −0.107, *P* = 0.641).

Low structuring among populations shown by microsatellite data could be due to male driven gene flow among populations, or ancestral polymorphisms if time is still insufficient for reciprocal monophyly [51]. In order to test if migration is currently taking place among populations and reject the possibility of ancestral polymorphisms, migration rates estimated in MIGRATE-N supported current migration among all populations in *T. inunguis* (M_2_), and among geographically isolated populations of *T. manatus* (M_3_), and rejected the model of no migration (M_0_) for both species (Table 4). Although these models with microsatellite data do not allow for a distinction of sex-biased dispersal, these results are in contrast with structure indices for mitochondrial data, pointing to male-driven gene flow as an explanation for the lack of structure in microsatellite markers.

**Table 4:** Migration models for *T. inunguis* and *T. manatus* ranked according to the harmonic mean of the likelihood, and Bayes factors for every model vs. that with the highest likelihood.

	Harmonic mean of all loci	Bayes factors
*T. inunguis*		
M2	−12874	
M3	−14620	3492
M1	−36179	46611
M0	−168836	311924
*T. manatus*		
M3	−50962	
M1	−82139	62355
M2	−140693	179464
M4	−838113	1574302
M0	−2254637	4407351

## Discussion

Riverine South American manatees inhabit dark waters, which have made it difficult for field experts to understand their population dynamics. This multi-locus approach enabled the study of phylogeographic patterns and population genetics, opening a window to understand the current status of these populations and their levels of connectivity.

### Phylogeographic Patterns

A complex cluster of connected haplotypes for *T. inunguis* suggests a rapid divergence from an ancestral population, with low but significant structure between Colombian and Peruvian populations. Colombian Amazonian populations lack structuring when compared to Brazilian *T. inunguis* manatees, implying two big Amazonian manatee populations, a western-most population, which includes samples from Peruvian Amazonian rivers, and an easterner population, which includes samples from Colombian and Amazonian rivers in Brazil. This division could be caused by isolation by distance, and historical geographic patterns, while interconnection among manatee within Western and Eastern populations may be due to flood pulse [52], where individuals inhabit smaller tributaries and floodplains during the high water season and migrate to the main rivers during the low water season [8]. This pattern has also been proposed for other Amazonian aquatic vertebrates such as the tambaqui (*Colossoma macropomum*), tucuxi (*Sotalia fluviatilis*) caiman (*Caiman crocodilus*), and the pirarucu (*Arapaima gigas*), among others [53], [54], [55], [56]. Further studies on the evolutionary history of Amazonian manatees using a broader set of molecular markers is needed to allow for more accurate molecular dating, leading to a more robust phylogeographic hypothesis regarding the origin and diversification of this species.

High genetic diversity was found for both species according to *D-loop* analysis (Table 1). *T. manatus* populations showed high levels of structuring between Sinú, San Jorge and Magdalena rivers. Structure was also found within rivers, with high differentiation between Northern Magdalena and Magdalena’s Ciénaga de Paredes (Table 3). The structure found among Northern Magdalena, San Jorge and Magdalena River’s Ciénaga de Paredes was not expected, as they seem to lack any obvious physical or geographic barriers, supporting the female philopatry hypothesis. Regardless of the small sampling size, this is preliminary evidence of population structuring between the Orinoco and Meta rivers, further analysis increasing sampling sizes for these rivers are needed.

### Sex-biased Gene Flow Dispersal

If females were dispersing, lack of structure between populations would be expected when analyzing mitochondrial and microsatellite data, while male dispersal and female philopatry would show population structure for mitochondrial data, but none or lesser levels of structure for microsatellites data, as has been reported for other aquatic mammals (i.e., Amazon river dolphin [*Inia geoffrensis*], Hollatz *et al.* 2011; sperm whales [*Physeter macrocephalus*], Lyrholm *et al.* 1999; dusky dolphins [*Lagenorhynchus obscurus*], Cassens *et al.* 2005; and Dall’s porpoise [*Phocoenoides dalli*], Escorza-Treviño & Dizon 2000 [57], [58], [59], [60]). Microsatellite data presented high homozygote frequencies for all populations of *T. manatus* and *T. inunguis*, which supported the presence of null alleles and deviations from HWE. However this pattern could be caused by other factors such as inbreeding, Wahlund effect (as a result of substructure within sampled populations) or shared ancestral polymorphisms. Our results suggest there are more likely explanations [61]: If null alleles were present, the pattern expected would be homozygote excess within and across all populations [61]. If this pattern is sustained across loci within populations, as was observed for populations of *T. manatus* and *T. inunguis*, the null allele hypothesis is rejected and the possibilities of other factors that explain low *H_O_* cannot be dismissed. Null migration among populations was the least supported migration model dismissing the possibility of ancestral polymorphisms as a force generating lack of structure among populations (Table 4). MBD produces mixture of populations, as males sampled could either be resident or immigrants, giving support to Wahlund effect as the main factor driving heterozygote deficit of South America riverine manatee populations [62].

*F_ST_* pairwise distances of microsatellite loci and *D-loop* were not correlated, with microsatellite data revealing gene flow between almost all populations per species. High support for full symmetric migration model among all *T. inunguis* populations, and low significant structure between Colombian and Peruvian Amazon contrast with higher levels of *D-loop* structure found for *T. manatus* populations, giving support to the Amazonian flood pulse as a strong force connecting populations, promoting gene flow among them.

The migration model supported for *T. manatus* is consistent with geographic barriers among sampled populations, where Meta and Orinoco rivers are isolated from Sinú, San Jorge and Magdalena rivers. These results support the migration of male manatees between the San Jorge and Magdalena rivers, and between the Magdalena and Sinú rivers through the Caribbean coast of Colombia; the area between these rivers consists of seacoasts and wetland systems [63], which could provide freshwater sources for males to successfully migrate. We attempted using chromosome Y introns (DBY7, TML-SMCY and SMCY17) [64], [65] to address the MBD hypothesis. However they did not provide useful information due to ancestral shared polymorphism among species revealing no polymorphisms. For further analysis we recommend developing Y linked microsatellite loci [66]. These male-dispersal movements between adjacent populations should be tested through the use of GPS satellite-linked radiotelemetry.

The low diversity found for microsatellite data was congruent with previously reported estimates of diversity for West Indian manatees in Belize, Mexico, Puerto Rico, and Florida. However, marine West Indian manatees presented population structure contrasting with gene flow in riverine populations [19], [20], [21] We suggest that habitat differences between marine and riverine environments impose different barriers to male dispersal, on which marine *T. manatus* male migrations are limited by freshwater resources to avoid osmoregulation stressors [67], strong currents, and presence of seagrass beds which restricts them from dispersing across deep waters [68], [69], while riverine males are mostly limited by strong currents and water levels [8], [10], being able to move for longer distances, relying more on behavioral, acoustic or chemical signals to pursue females [4].

### Conservation Implications

The Amazonian and the West Indian manatees are listed as vulnerable by the International Union for the Conservation of Nature and Natural Resources (IUCN) since 1982 [70], and Appendix I of the Convention on International Trade in Endangered Species of Wild Fauna and Flora (CITES) since 1975, with populations severely affected by habitat degradation and intense hunting in the past centuries [6], [25], [71], [72], [73].

Population structure detected in riverine West Indian and Amazonian manatees when using maternally inherited *D-loop* should be taken into account by current reintroduction programs, being important to release animals on their place of origin, even if males are dispersing among populations, as previously proposed by Vianna *et al.* 2006. High *D-loop* haplotype diversity of South American riverine manatees reveals that river dynamics could be key in maintaining population’s genetic diversity.

Conservation strategies in South American manatees should protect riverine populations not only locally, but also on a wider scale, given this is critical in maintaining connectivity among populations. This includes connectivity between coastal wetland systems between the Magdalena and Sinú rivers in the Caribbean coast of Colombia so as to encourage the use of aquatic ecological corridors by male *T. manatus* between these two populations, to warrant male gene flow and reducing the chances for inbreeding.

## Supporting Information

R Script S1 **R script used for microsatellite allele randomization.**
(R) Click here for additional data file.

R Script S2 **R script used for microsatellite locus bootstrapping.**
(R) Click here for additional data file.

R Script S3 **Assessment of population monophyly on each genealogy.**
(R) Click here for additional data file.
